# Limited Effect of Y Chromosome Variation on Coronary Artery Disease and Mortality in UK Biobank—Brief Report

**DOI:** 10.1161/ATVBAHA.122.317664

**Published:** 2022-07-14

**Authors:** Paul R.H.J. Timmers, James F. Wilson

**Affiliations:** MRC Human Genetics Unit, MRC Institute of Genetics and Cancer (P.R.H.J.T., J.F.W.), University of Edinburgh, United Kingdom.; Centre for Global Health Research, Usher Institute (P.R.H.J.T., J.F.W.), University of Edinburgh, United Kingdom.

**Keywords:** blood pressure, chromosomes, human, Y, coronary artery disease, genetic variation, hypertension, mortality

## Abstract

The effect of genetic variation in the male-specific region of the Y chromosome (MSY) on coronary artery disease and cardiovascular risk factors has been disputed. In this study, we systematically assessed the association of MSY genetic variation on these traits using a kin-cohort analysis of family disease history in the largest sample to date.

**Methods:**

We tested 90 MSY haplogroups against coronary artery disease, hypertension, blood pressure, classical lipid levels, and all-cause mortality in up to 152 186 unrelated, genomically British individuals from UK Biobank. Unlike previous studies, we did not adjust for heritable lifestyle factors (to avoid collider bias) and instead adjusted for geographic variables and socioeconomic deprivation, given the link between MSY haplogroups and geography. For family history traits, subject MSY haplogroups were tested against father and mother disease as validation and negative control, respectively.

**Results:**

Our models find little evidence for an effect of any MSY haplogroup on cardiovascular risk in participants. Parental models confirm these findings.

**Conclusions:**

Kin-cohort analysis of the Y chromosome uniquely allows for discoveries in subjects to be validated using family history data. Despite our large sample size, improved models, and parental validation, there is little evidence to suggest cardiovascular risk in UK Biobank is influenced by genetic variation in MSY.

**Highlights**

- Genetic variation in the Y chromosome is highly structured across Great Britain, warranting adjustments for population structure and geographic confounding when investigating its link to disease.
- Putative associations of Y haplogroups with disease in subjects can be validated using kin-cohort analysis, where father disease acts as replication and mother disease acts as negative control.
- Among 152 186 White British individuals from UK Biobank, none of the tested Y haplogroups are meaningfully associated with coronary artery disease, its risk factors, or all-cause mortality.
- Statistically suggestive Y haplogroup associations in UK Biobank subjects are not supported by parental data.

The Y chromosome is the smallest human chromosome spanning only 57.2 Mb (23 Mb euchromatic) and containing just ≈78 protein-coding genes.^1^ Its sequence can be classified into 2 parts: (1) the pseudoautosomal regions, which constitute around 5% of its sequence, and (2) the male-specific region of the Y chromosome (MSY). Although the pseudoautosomal regions are homologous to the X chromosome, the MSY is unpaired and therefore does not undergo recombination.^2^ The lack of MSY recombination allows this region to be transmitted largely unchanged from father to son, and as such, it can be used to trace patrilineal ancestry^3^ and migration.^4^

Despite its small size, the Y chromosome has been hypothesized to account for sex differences in coronary artery disease (CAD) susceptibility.^5^ Since 2000, a series of small-scale studies investigated the link between blood pressure and the *Hin*dIII(–) polymorphism—known as the P haplogroup using modern notation^6^—for individuals from various European ancestries. These studies found the P haplogroup was associated with higher blood pressure^5^ (N=409), lower blood pressure^7^ (N=920), or no change in blood pressure^8–10^ (N=2743; N=1983; N=2700). Similarly, the P haplogroup has been linked to higher levels of low-density lipoprotein cholesterol and triglycerides in one study^11^ (N=1288) but showed no such effect in another^9^ (N=1983). Other polymorphisms, such as those in Y chromosome genes *TBL1Y* and *USP9Y*, have been linked to changes in high-density lipoprotein cholesterol and triglyceride levels only in black individuals.^12^

The largest MSY study to date was performed by Eales et al^13^ (2019), who found an association between CAD incidence in UK Biobank (N_cases_=11 234; N_controls_=117 899) and the I1 haplogroup (I1-M253) after correcting for cardiovascular risk factors and family history of heart disease.^13^ Despite the increased sample size, 3 methodological concerns draw into question their findings.

First, recent studies show uncorrected fine-scale population structure in UK Biobank causes geographic and socioeconomic confounding that results in spurious associations with disease traits^14–16^ This issue may be especially pertinent for the MSY, which is uniquely sensitive to population structure.^17,18^ Eales et al^13^ only corrected for 5 genetic principal components, which may not be adequate to rule out such confounding. Second, cardiovascular risk factors are heritable and can therefore cause collider bias when used as covariates.^19^ More precisely, MSY haplogroups with causal effects on risk factors (but not CAD directly) can have biased associations with CAD after adjusting for the risk factor. Figure S1 illustrates this scenario in more detail. Third, correcting for paternal history of heart disease in an MSY study of CAD is inappropriate since fathers and sons share the same MSY. This adjusted estimate captures the excess effect of MSY haplogroups on CAD in the current generation compared to their effect on heart disease in the previous generation.

Given these conflicting results and methodological concerns, a reexamination of the role of genetic variation in MSY on cardiovascular risk and disease outcomes is warranted. Here, we systematically attempt to validate findings from previous studies using the latest release of medical records from UK Biobank. We mitigate concerns about collider bias and population stratification by replacing heritable risk factors with adequate genetic principal components and geographic covariates. Instead of adjusting for family history traits, we use information on parental disease to perform a special type of kin-cohort analysis,^20^ where we test UK Biobank participant MSY haplogroups against paternal traits as validation and maternal traits as negative control. In contrast to previous studies, we conclude there is limited evidence for an association of MSY haplogroups with cardiovascular disease phenotypes and all-cause mortality.

## MaterialS and Methods

### Availability of Data and Material

UK Biobank phenotypes and genotypes are available upon application (https://www.ukbiobank.ac.uk/).

The 2011 census UK area boundaries and Townsend deprivation data are publicly available from the UK Data Service and can be accessed at http://dx.doi.org/10.5257/census/aggregate-2011-2. Prevalence maps of the 90 MSY haplogroups by area of birth for UK Biobank men have been made publicly available at the Edinburgh DataShare and can be accessed at https://doi.org/10.7488/ds/3472. The yhaplo software used to infer MSY haplogroups is publicly available on GitHub and can be accessed at https://github.com/23andMe/yhaplo. The statistical code used to generate the results in this study has been made publicly available on GitHub and can be accessed at https://github.com/PaulTimmers/ATVB-MSY.

### Data Sources and Quality Control

All analyses were performed using UK Biobank samples.^21,22^ Around half a million individuals aged 40 to 69 years were recruited between 2006 and 2010 and completed touchscreen questionnaires and verbal interviews regarding their own health and that of their parents. Electronic health record linkage included hospital admissions, surgical procedures, and death records covering the period of 12 December 1980 to 12 November 2021. The majority of participants were genotyped using the UK Biobank Axiom array, with around 50 000 individuals genotyped on the mostly overlapping UK BiLEVE array. Participant sex was both self-reported and inferred from X and Y chromosome marker intensities, as described in Bycroft et al.^22^

### Identification of Y Haplogroups

Phylogenetic analysis was performed on all male-inferred UK Biobank participants (N=223 513) using yhaplo v1.1.2.^23^ Among the 13 569 reference SNPs (single nucleotide polymorphisms) provided by yhaplo, 232 were genotyped on both UK Biobank arrays. SNPs with a call rate of <95% (n=39) and monomorphic SNPs (n=27) were removed, leaving 166 SNPs for haplogroup inference. Eighty-nine haplogroups were reported directly by yhaplo, and a further 38 were defined through combinations of derived groups to increase statistical power of rarer haplogroups and to detect effects arising from deeper nodes in the Y chromosome genealogy (Table S1). For example, G-M201 was created from all individuals carrying the 9 derived G haplogroups, analogous to combining the effects of these individual haplogroups. In a few cases, these newly defined haplogroups were approximate, as with R1b-S21, where 4 derived subgroups could be recognized from the SNPs available on the array, other members of this group being subsumed into the upstream paragroup (in this case R1b-S128*). Haplogroups were named using up to 5 characters of the hierarchical nomenclature from the International Society of Genetic Genealogy 2019-2020 tree (https://isogg.org/tree/; eg, J1a2b), followed by the SNP name defining the haplogroup (eg, J1a2b-L817). Paraphyletic groups or paragroups (where the group does not include every descendant of the ancestor) are indicated with an asterisk suffix (eg, R1b-S191*, which does not contain the descendant groups R1b-S3787 and R1b-FGC15498). All the main deep branches of the Y haplogroup tree (eg, G, H, I, J, N, each of which represents tens to hundreds of coinherited SNPs^24^) were recovered in this way, with a large, but random selection of more recent and rarer haplogroups, the defining SNPs for which happened to be present on the genotyping arrays.

### Cardiovascular Phenotype Definitions

At assessment, systolic blood pressure (SBP) and diastolic blood pressure (DBP) were measured using an automated OMRON digital sphygmomanometer. If multiple technical replicates were available, the mean value was used. Analogous to Eales et al,^13^ we discarded SBP and DBP outliers based on a modified Tukey test: values >3 interquartile range below the 25th percentile or >3 interquartile range above the 75th percentile. The interquartile range was recalculated after removing outliers, and the test was repeated until no further values were removed. Mean arterial blood pressure was calculated as DBP+(DBP+SBP)/3. As is standard for genetic analyses of blood pressure,^25,26^ individuals who reported taking antihypertensive medications had their SBP increased by 15 mm Hg and their DBP increased by 10 mm Hg. Individuals taking antihypertensive medication or showing either SBP ≥140 or DBP ≥90 were classified as cases of hypertension.

Lipid levels were measured at assessment using a Beckman Coulter AU5800 clinical chemistry analyzer. Total cholesterol and triglycerides were measured by glycerol-3-phosphate peroxidase analysis, whereas high and low-density lipoprotein cholesterol were measured by enzyme immunoinhibition analysis and enzymatic protective selection analysis, respectively. As is standard for genetic analyses of lipid levels,^26,27^ individuals who reported taking lipid-lowering medication had their low-density lipoprotein cholesterol and total cholesterol measures divided by 0.7 and 0.8, respectively. After log-transformation of triglyceride values, all lipid phenotypes had outliers removed using the modified Tukey test. Again, outlier removal was repeated until no further values were removed.

CAD was defined using the classification algorithm proposed by Eales et al,^13^ which uses participant reports coded by the interviewing nurse or doctor, hospital, and death records coded using the *International Classification of Diseases (ICD*) chapters and surgical records coded using the OPCS (Office of Population Censuses and Surveys) classification system. In brief, the algorithm defines CAD cases by the presence of self-reported or hospital-recorded incidence of myocardial infarction (*ICD, Ninth Revision* codes 4109, 4129; *ICD, Tenth Revision* codes I21–I23, I25.2), percutaneous coronary intervention (OPCS codes K49, K50, K75), or coronary artery bypass graft (OPCS codes K40–46). In addition, individuals with death records showing CAD (*ICD, Tenth Revision* codes I20, I21, I24, I25.1, I25.2, I25.5, I25.8, I25.9) as a primary or secondary cause of death were also defined as cases. Controls were defined as all remaining individuals who did not report or had no hospital records of (unstable) angina (*ICD, Ninth Revision* code 4139; *ICD, Tenth Revision* codes I20.0, I20.1, I20.8, I20.9) and were not taking any medications containing aspirin, glyceryl trinitrate, isosorbide mono- or dinitrate, or nicorandil (see Table S2 for medication codes). Among the unrelated male British UK Biobank participants, the algorithm identified 16 223 cases and 129 533 controls (after excluding 23 733 individuals).

Father and mother hypertension and CAD were defined using self-reported data from subjects. Specifically, a touchscreen questionnaire asking “Has/did your father ever suffer from? (You can select more than one answer),” allowed subjects to select whether their father suffered from a list of disorders including heart disease and high blood pressure. An analogous question was asked about their mother’s illnesses. Father and mother hypertension cases were defined by the selection of high blood pressure by the participant, whereas CAD cases were defined by the selection of heart disease (the accuracy of heart disease as a proxy for CAD is assessed below). All other selections were denoted as controls unless the participant preferred not to answer. Finally, to gather more data on the association of IJK-S137 with higher blood pressure, we selected subjects with only one male sibling, and created brother hypertension variables from the question “Have any of your brothers or sisters suffered from any of the following diseases? (You can select more than one answer),” analogous to father and mother hypertension definitions.

### Socioeconomic Deprivation Variables

Spatial coordinates and deprivation indices were retrieved from the UK Office for National Statistics 2011 census.^28^ North and east coordinates of UK Biobank participant postcodes (rounded to the nearest kilometer) were projected onto the European Petroleum Survey Group Projection 27700 reference and then mapped onto geographic regions (eg, Lower Super Output Areas) using R packages sp v1.4-5 (Pebesma and Bivand,^29^ Bivand et al^30^) and rgdal v1.5-23 (Bivand et al^31^). This mapping was done for the first instance of (1) home location coordinates at assessment and (2) birthplace coordinates.

### Statistical Analysis

All statistical analyses were performed using R 3.6.1, unless otherwise specified.

#### Linear Regression

Associations with blood pressure and lipid traits were tested using a multivariable linear model, implemented in the R package speedglm v0.3-3 (Enea^32^). The presence or absence of a Y haplogroup was coded as a binary variable, and each haplogroup with at least 100 samples was tested in a separate model (90 tests total). The following model was used:


Y=βX+γZ+∈ (1)

where *Y* is an N×1 vector of reported blood pressure or lipid trait values for N individuals, *X* is an N×M matrix of M covariates, β is the M×1 vector of covariate coefficients, and *Z* is an N×1 vector of binary indicator values for the selected haplogroup. The estimate of interest, γ denotes the selected haplogroup coefficient.

#### Logistic Regression

Associations with (parental) CAD and hypertension were tested using a multivariable logistic model, implemented in the R package speedglm v0.3-3 (Enea^32^). The following model was used:

$$P(Y)\;=\frac{1}{1\;+\;e^{-(\beta X\;+\;\gamma Z\;+\;\in )}}$$ (2)

where *P*(*Y*) is the probability of participant CAD or hypertension. The other variables are as in Equation 1. Separate models were fit for each haplogroup containing at least 40 cases of CAD (66 total), or 100 individuals with complete blood pressure data (90 total).

#### Cox Proportional Hazards Regression

Associations with (parental) all-cause mortality were tested using a Cox proportional hazards model, implemented in the R package survival v3.2-11 (Therneau et al^33^). The following model was used:


$$h(x)=h_{0}(x)e^{\beta X+\gamma Z+\in }$$ (3)

where h(x) is the hazard at age x, h_0_(x) is the baseline hazard at age x, and the remaining variables are as described in Equation 1.

Survival models were constructed using participant age at assessment, with death status inferred from the presence of a death record at time of analysis. The censoring date was set to 01 November 2021 to allow for a 2-week delay in the processing of death records (ie, the latest recorded deaths occurred mid-November 2021, but there may be deaths from the start of November that have not been registered yet). Parental survival models were constructed using the parent age and alive/dead status as reported by the participants at time of assessment. Parents with reported age at death before 40 were excluded to limit deaths due to accident or injury (6816 father exclusions, 5070 mother exclusions).

#### Covariates

The Eales et al^13^ models were adjusted for the continuous variables, age, body mass index, and the first 5 genetic principal components, as well as the categorical variables, genotyping array (2 levels), hypertension (2 levels), number of days performing moderate exercise (8 levels), average household income (5 levels), smoking history (2 levels), completion of further education (2 levels), employment status (6 levels), weekly alcohol intake (6 levels), father CAD (2 levels), and mother CAD (2 levels). Exact definitions of each variable can be found in Eales et al.^13^ Outliers in the age and body mass index phenotypes were removed based on the modified Tukey test, as described above.

Socioeconomically robust models were adjusted for the continuous variables, (parent) age, (parent) age squared, north and east coordinates at assessment and birth, Townsend deprivation index of the Lower Super Output Area at assessment and birth, and the first 40 principal components of ancestry, as well as the categorical variable, genotyping array (2 levels). As survival models already incorporate (parent) age to calculate survival, linear and quadratic age were not used as covariates in the survival model.

We include models adjusting only for age (if appropriate), 40 principal components, and genotyping array, without further adjustment for geography or socioeconomic variables as sensitivity analyses in Supplemental Tables S5 to S9.

#### Significance Threshold

There is substantial correlation between Y haplogroups because of their hierarchical nature; therefore, adjusting for multiple comparisons using Bonferroni correction is too stringent. Principal component analysis of the correlation matrix was used to identify the number of independent components explaining 95% of the variation in Y haplogroups. This identified 22 independent components for the 66 Y haplogroups used in the CAD model, and 38 components for the 90 Y haplogroups used in the other models. Separately, we also estimated the number of components for related blood pressure traits (2 components) and lipid traits (2). For each phenotypic category, *P* values were adjusted by multiplying them by the number of independent haplogroups and, if applicable, the number of independent phenotypes.

### Accuracy of Heart Disease as a Proxy for CAD

Heart disease describes a variety of conditions affecting the heart, of which CAD is only a subset. The proportion of parental heart disease cases meeting CAD inclusion criteria was calculated using father-son pairs in UK Biobank. As such pairs are not directly reported by UK Biobank, father-son relationships were inferred from autosomal genetic relatedness and subject-reported age of fathers alive at assessment. Specifically, for each unrelated White British subject in UK Biobank, father age (in years) was subtracted from the date of subject assessment, creating a father date of birth interval allowing for 6 months on either side. Pairs of UK Biobank men with a genetic kinship coefficient >0.2 and containing one date of birth covered by the other’s father's date of birth interval were inferred to be father-son pairs (n=301). For each father, CAD was inferred using the Eales et al^13^ algorithm described above. CAD proxy accuracy was calculated as the proportion of CAD cases among fathers reported to have heart disease by their sons (CAD cases=26 and heart disease cases=37). CIs were approximated by bootstrapping all father-son pairs and recalculating this proportion (100 000 iterations).

## Results

**Figure 1.**
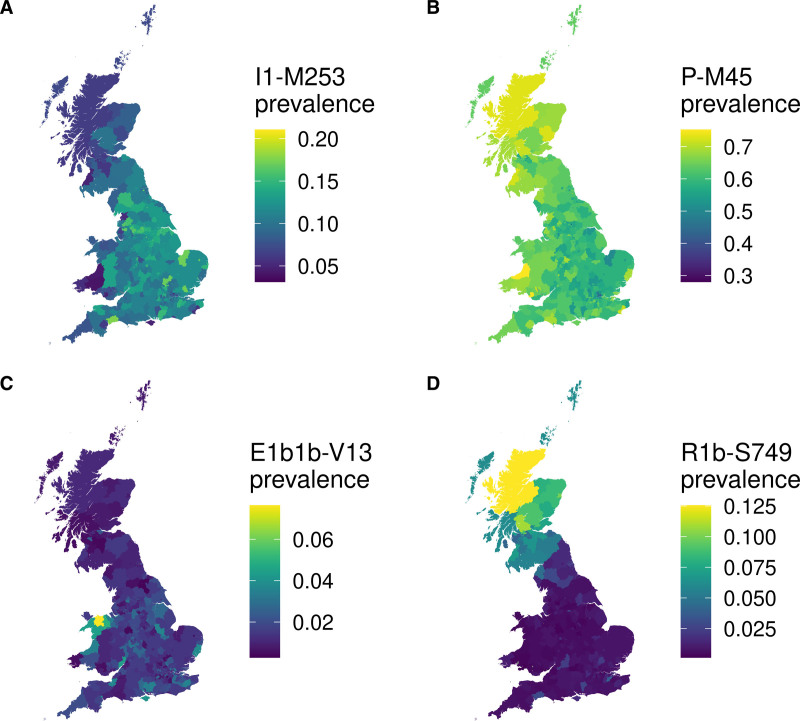
**Examples of geographic structuring of Y chromosome variation by place of birth of genetically British men from UK Biobank.** The prevalence of male-specific region of the Y chromosome haplogroups was plotted by place of birth in successively larger areas with at least 100 individuals, from wards and electoral divisions, to local authorities, to regions of England and the nations of Great Britain. Displayed are examples with pronounced structuring (**A**) I1-M253 (**B**) P-M45, (**C**) E1b1b-V13, (**D**) R1b-S749. Note the prevalence scales are different between haplogroups. See https://doi.org/10.7488/ds/3472 for maps of all 90 haplogroups.

Genealogical analysis of 166 genotyped SNPs of the MSY of unambiguously male, unrelated UK Biobank individuals (N=223 566) using yhaplo^23^ successfully identified 103 unique Y haplogroups for 223 513 individuals. In line with known population genetic structure,^24^ Y haplogroups differed significantly by self-reported ancestry. For example, the most abundant haplogroups in self-reported British-, Chinese-, and African-heritage individuals were R-P311 (n=94 581; 47.8%), O-M122 (n=311; 55.0%), and E-M180 (n=1284; 77.8%), respectively. We restricted all subsequent analyses to unrelated individuals with genomically similar White British ancestry (n=169 635) to avoid confounding due to the extreme population stratification of Y chromosome variation.^17^ In addition to the Y haplogroups identified by yhaplo, we further grouped Y haplogroups hierarchically and kept for analysis only Y haplogroups and groupings with at least 40 cases of CAD or 100 individuals with nonmissing blood pressure and lipid trait values, for a total of 90 Y haplogroups (Table S1). As expected, the Y chromosome haplogroups carried by White British UK Biobank individuals show strong geographic structuring across the nations of Great Britain, with certain lineages being more common in England, Wales, or Scotland (see https://doi.org/10.7488/ds/3472 for all maps).^34^ For example, the I1-M253 and P-M45 haplogroups each show a latitudinal and longitudinal gradient, with the former increasing in prevalence towards England and the latter increasing in prevalence towards Scotland and Wales (Figure 1A and 1B). Even more pronounced are E1b1b-V13 and R1b-S749, which show region-specific frequency peaks in Gwynedd, North Wales, and northern Scotland, respectively (Figure 1C and 1D).

### I1-M253 Haplogroup Has No Detectable Effect on CAD

We first set out to replicate the CAD analysis in Eales et al.^13^ Using identical definitions of CAD and CAD risk factors, we identify 12 226 cases of CAD with complete covariate information in our UK Biobank sample, representing an increase of 1032 (9.2%) cases since the publication of their study (see Table S3 for sample descriptives). Fitting an identical logistic model to Eales et al,^13^ we find the I1-M253 haplogroup has a largely attenuated effect on participant CAD in our study (odds ratio, 1.06 [95% CI, 1.00–1.12]; *P*=0.058) compared with the original estimate (1.11 [95% CI, 1.04–1.18]; *P*=7×10^–4^; Figure S2; Table S4A). Moreover, increasing the number of genetic principal component covariates from 5 to 40 to account for any residual population stratification further attenuates this effect (1.05 [95% CI, 0.99–1.11]; *P*=0.104), with principal components 9 and 14 being significant confounders with moderate effects (95% CI, 1.01–1.02; each *P*<5×10^–4^; Table S4B). Simplification of this model by removing father CAD as a covariate—which is also dependent on the participant haplogroup—further reduces the strength of the association (1.04 [95% CI, 0.99–1.11]; *P*=0.141), despite the increased sample size with complete covariates (N_cases_=12 424; N_controls_=105 231; Table S4C).

### Y Haplogroups Are Not Associated With CAD or All-Cause Mortality

Next, we set out to systematically assess all Y haplogroup associations with CAD, hypertension, and mortality using new models. We did not include heritable covariates in our models to avoid collider bias^35^ and added geographic/deprivation variables and 40 principal components to mitigate confounding due to geography and socioeconomic differences.^15^ Finally, our models included a quadratic age term to account for any nonlinear associations of cardiovascular phenotypes with age.^36^ This more restricted set of covariates allows the dependent variable in our model to be replaced by reported father disease or death (as validation), and mother disease or death (as negative control), assuming geographic/deprivation variables are similar for participants and their parents.

**Figure 2.**
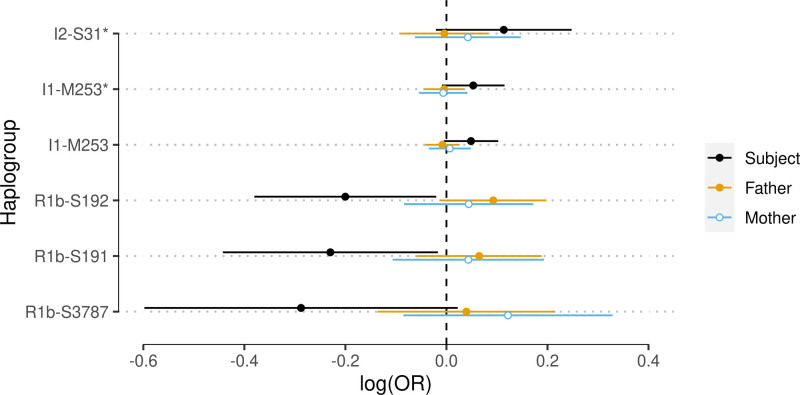
**Parental validation does not support putative male-specific region of the Y chromosome (MSY) haplogroup associations with subject coronary artery disease (CAD).** Shown are the 6 MSY haplogroups showing some suggestive association with subject CAD (*P*<0.10) in models adjusted for socioeconomic and geographic variables (out of 66 haplogroups tested). Effect estimate units are log odds ratios for CAD (subjects) or heart disease (parents). Lines around point estimates represent 95% CIs. See Table S5 for all MSY haplogroups. OR indicates odds ratio.

In our models, we find no evidence for any of the 66 MSY lineages associating with CAD at the Bonferroni-adjusted significance threshold of *P*<0.0023. Kin-cohort validation of Y haplogroups reaching suggestive significance (*P*<0.10) shows MSY haplogroup effects on CAD suggested by subjects are not directionally consistent with MSY haplogroup effects on father heart disease (Figure 2; Table S5). Parental validation also does not support the suggestive effect observed for I1-M253. As heart disease is an imperfect proxy for CAD (between 55% and 85% of cases of father heart disease are expected to meet CAD inclusion criteria in our study), power to replicate CAD-specific effects in parents may be reduced (although power to detect heart disease more generally is unaffected). Performing the same analysis on hypertension and separately, all-cause mortality, we again find no robust evidence for an association with any of the haplogroups (all *P*>0.0013; Table S6; Table S7). Here, haplogroups reaching suggestive significance (*P*<0.10) have no effect in fathers and/or overlap with the maternal negative control (Figure S3, Figure S4).

### Y Haplogroup Associations With Blood Pressure and Lipid Traits

When testing 90 Y haplogroups against blood pressure traits in participant models corrected for socioeconomic status, we find only the IJK-S137 haplogroup is significantly associated with increased blood pressure compared to the other haplogroups. The largest effect of this haplogroup is on mean arterial pressure, which is associated with an increase of 0.93 mm Hg (95% CI, 0.46–1.41 mm Hg; *P*=0.0001; *P*_adj_=0.009; Figure S5; Table S8). Notably, the IJK-S137 haplogroup is not significantly associated with an increase in participant or father hypertension, although the direction of effect is concordant. Testing the same group against brother hypertension (N_cases__=_3321; N_controls__=_21 391), we again find a directionally concordant effect, but we lack power to confirm the association (Figure S6). In contrast, we find no evidence for an effect of any Y haplogroup on cholesterol levels or triglycerides (*P*>6.58×10^–4^; Table S9).

## Discussion

Genetic variation in the human Y chromosome has been hypothesized to influence cardiovascular risk in men. We performed a comprehensive MSY haplogroup association analysis of cardiovascular risk factors, CAD, and all-cause mortality in unrelated, genomically British individuals from UK Biobank. We find no evidence for an effect of any MSY haplogroup on subject CAD or hypertension, and parental models do not support statistically suggestive associations identified in subjects.

We do detect a statistically significant effect of the IJK-S137 haplogroup on measures of blood pressure in UK Biobank subjects but cannot validate this association using parental models as we lack parental blood pressure measurements. Instead, we find directionally consistent effects of this haplogroup on hypertension across male kin, although we lack the power to reliably confirm the association. In any case, the small effect of IJK-S137 on blood pressure (<1 mm Hg) leads us to hypothesize its effect on hypertension risk—if real—is likely clinically insignificant.

Despite our large sample, we did not replicate previously reported associations between cardiovascular phenotypes and the P-M45,^5,7,11^ I-M170,^37^ or I1-M253 haplogroups.^13^ It is possible findings from these studies were false positives due to their smaller sample sizes (ie, greater sensitivity to outliers), less well-controlled population stratification, collider bias, or geographic confounding. However, differences in haplogroup frequency between studies complicate direct comparisons of haplogroup effect estimates. We can confidently compare our effects with those reported by Eales et al^13^ as we replicated their UK Biobank sample, but haplogroup frequencies in other European cohorts used in previous studies will be different from genomically British UK Biobank individuals. As such, there could be population-specific effects of MSY haplogroups that are not apparent in UK individuals.

The MSY haplogroup kin-cohort analysis we present is a useful framework for validating putative associations of phenotypes with genetic variation in the Y chromosome and can be easily applied to any family history phenotype of interest. However, this method relies on several assumptions. First, we made the simplifying assumption that kin effect size estimates are independent. Strictly, phenotypic correlations between family members due to shared environment will result in MSY haplogroup effect size correlations. Although this can be adjusted for by inflating standard errors,^38^ we saw no need to perform the adjustment as we did not find any significant kin effects to begin with.

Second, using mothers as negative control assumes the participant Y haplogroup and their mother’s risk of CAD and hypertension are independent. It is conceivable this assumption could be violated if genetic variation in the participant indirectly influences the mother phenotype. For example, mothers are at increased risk of CAD after losing a child (especially if the cause of death was CAD),^39^ although it is difficult to disentangle this indirect effect from any shared environment and autosomal genetic confounders. Alternatively, the independence assumption could be violated if genetic variation inherited from the mother (ie, autosomal or mitochondrial) modifies Y haplogroup effects. Such epistatic effects exist between autosomal CAD variants,^40^ and it remains to be tested whether this phenomenon extends to genetic variation in the Y chromosome. We are underpowered to exclude the presence of indirect or epistatic correlations between MSY haplogroups and maternal phenotypes but expect this correlation to be a fraction of the MSY haplogroup effects on participants themselves.

Third, we did not test whether our results were influenced by mosaic loss of Y chromosome (mLOY). Older men and men with unhealthy lifestyles tend to experience a greater degree of mLOY.^41^ It is likely severe mLOY leads to poor genotype call rates, and as such, individuals with substantial mLOY could have been removed from our analysis, potentially resulting in a sample biased to be healthier than average. However, in an effort to reproduce the work by Eales et al,^13^ we did not take into account mLOY and used the same quality control criteria as their study, which should have allowed us to detect an effect with our sample if one were there.

Finally, it is important to note our analyses do not rule out all effects of the Y chromosome on human disease. Large structural variations such as Y chromosome deletions are known to have pronounced effects on male fertility and possibly cancer.^42^ Rather, we find no evidence of any common or rare MSY haplogroup lineages tested here to significantly influence CAD, hypertension, or all-cause mortality. It remains possible that other types of variation, such as epigenetic or transcriptomic differences, could influence CAD risk. Future work will involve testing the effects of rarer MSY haplogroups on CAD—possible when whole-genome sequences in large population biobanks become available—and testing the effects of MSY nucleotide variation on health and disease beyond cardiovascular phenotypes.

## Article Information

### Acknowledgments

The authors thank UK Biobank for making their data available through application 19655. Geographic maps and socioeconomic variables were derived from National Statistics data Crown copyright and database right 2022, NRS (National Records of Scotland) data Crown copyright and database right 2022, source: NISRA (Northern Ireland Statistics and Research Agency); website: www.nisra.gov.uk, and OS (Ordnance Survey) data Crown copyright and database right (2022). For the purpose of open access, the authors have applied a Creative Commons Attribution (CC BY) license to any Author Accepted Manuscript version arising from this submission. P.R.H.J. Timmers contributed to Conceptualization, Methodology, Software, Formal analysis, Investigation, Writing—Original Draft, Writing—Review & Editing, Visualization. J.F. Wilson contributed to Conceptualization, Methodology, Formal analysis, Investigation, Resources, Writing—Original Draft, Writing—Review & Editing, Supervision, Funding acquisition.

### Sources of Funding

The authors acknowledge funding from the Medical Research Council Human Genetics Unit program grant, Quantitative traits in health and disease (U. MC_UU_00007/10).

### Disclosures

P.R.H.J. Timmers is an employee of BioAge Labs, Inc. The other author reports no conflicts.

### Supplemental Material

Figures S1–S6

Tables S1–S9

## Supplementary Material


