# Supplementary material for: Limited Effect of Y Chromosome Variation on Coronary Artery Disease and Mortality in UK Biobank—Brief Report
Source: Arterioscler Thromb Vasc Biol. 2022 Jul 14;42(9):1198–206. doi: 10.1161/ATVBAHA.122.317664 (PMC9394501; doi:10.1161/ATVBAHA.122.317664)
Supplement: Supplementary file 2 [file atv-42-1198-s002.pdf]

SUPPLEMENTAL MATERIALS

Limited effect of Y chromosome variation on coronary artery disease and mortality in UK Biobank

Paul R.H.J. Timmers[1,2], James F. Wilson[1,2]

1) MRC Human Genetics Unit, MRC Institute of Genetics and Cancer, University of Edinburgh, Edinburgh, United Kingdom
2) Centre for Global Health Research, Usher Institute, University of Edinburgh, Edinburgh, United Kingdom

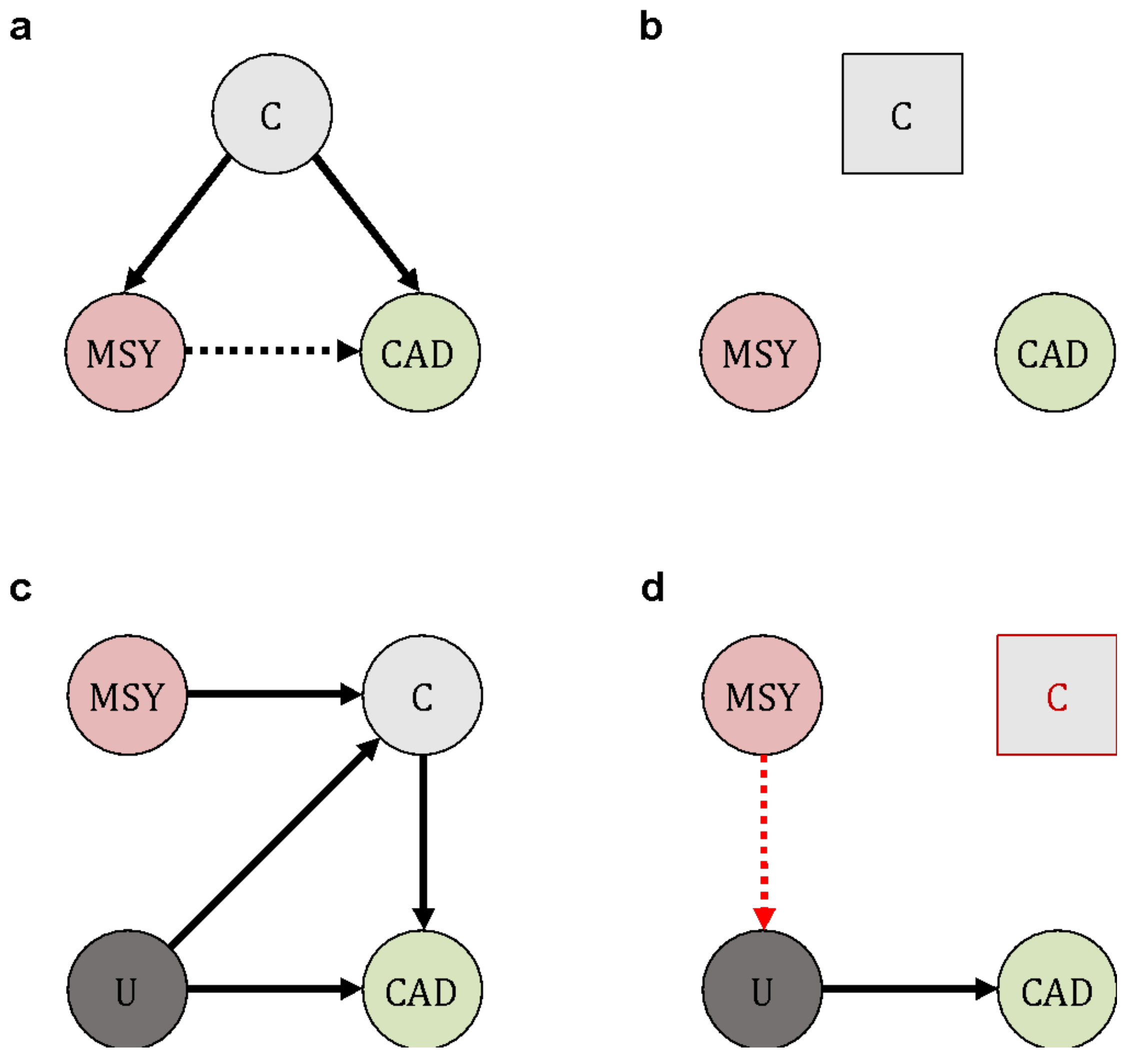

Figure S1: Directed acyclic graphs showing the effect of adjustment for (heritable) covariates on coronary artery disease (CAD). Circles represent phenotypes, squares represent conditioning on phenotypes, solid arrows represent causal effects, and dotted arrows represent non-causal associations. **a)** In a situation where the covariate (C) is independent from genetic variation (i.e., non-heritable), the male-specific region of the Y chromosome (MSY) haplogroup will not have a causal effect on the covariate, and adjustment for the covariate is appropriate. For example, if an MSY haplogroup and CAD are both more prevalent towards the north of the UK, a naïve measurement would show a false positive association between the two. **b)** Adjusting for a non-heritable covariate appropriately removes any association between the MSY haplogroup and CAD induced by the covariate. **c)** In a situation where the covariate is heritable, it is possible for the MSY haplogroup to have a direct causal effect on the covariate (and an indirect effect on CAD). Due to unmeasured (U) shared genetic and environmental confounders affecting both the covariate and CAD, multiple causal arrows now collide at the covariate (i.e., the covariate is a collider). **d)** Adjusting for a collider covariate opens a path between the MSY haplogroup and CAD through the unmeasured confounders (collider bias). The direction of this bias will depend on the direction of the causal effect of the MSY haplogroup on the covariate and the correlation between the covariate and CAD. For example, if the MSY haplogroup increases hypertension, adjusting for hypertension would remove the indirect effect of the MSY haplogroup on CAD though hypertension but could induce a new, negative association between the MSY haplogroup and CAD though the unmeasured confounders. Conversely, if the MSY haplogroup decreases hypertension, adjusting for hypertension could induce a new, positive association between the MSY haplogroup and CAD through the unmeasured confounders.

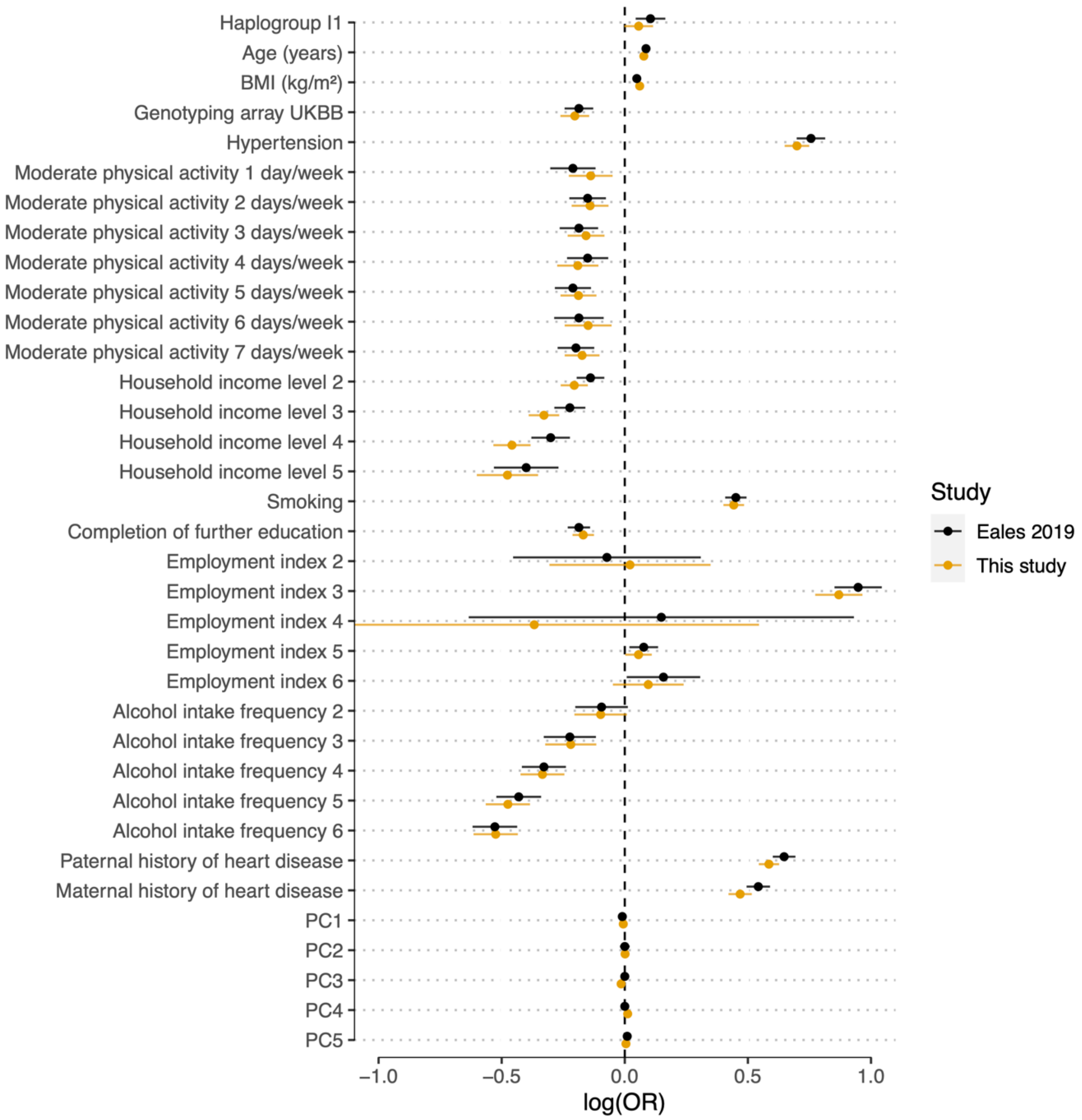

Figure S2: Replication of the Eales et al. 2019 model which identified an association between the I1 haplogroup and CAD. Shown are point estimates with 95% confidence intervals in log odds ratio units (logOR) of each of the variables on CAD in UK Biobank men. Variable definitions and exclusion criteria were similar between studies, although our study included more recent hospital and death records. The sample used in Eales et al. consisted of 11,234 cases and 117,899 controls. The sample used in our study consisted of 12,226 cases and 104,140 controls. BMI—Body mass index. PC—Principal components of autosomal genetic variation across the UK Biobank cohort.

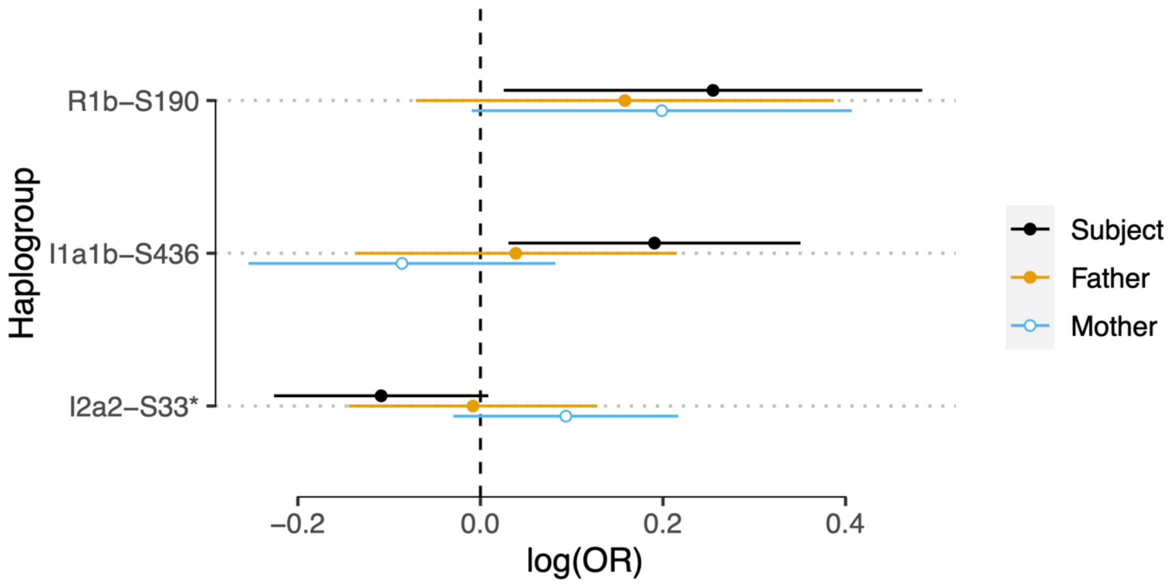

Figure S3: Parental validation of putative hypertension haplogroups. Shown are MSY haplogroups with suggestive associations with self-reported hypertension in White British UK Biobank subjects ($P < 0.10$). Units are log odds ratios (logOR), and lines around point estimates represent 95% confidence intervals. Mother hypertension is listed as a negative control. See **Table S6** for coefficients of all MSY haplogroups tested.

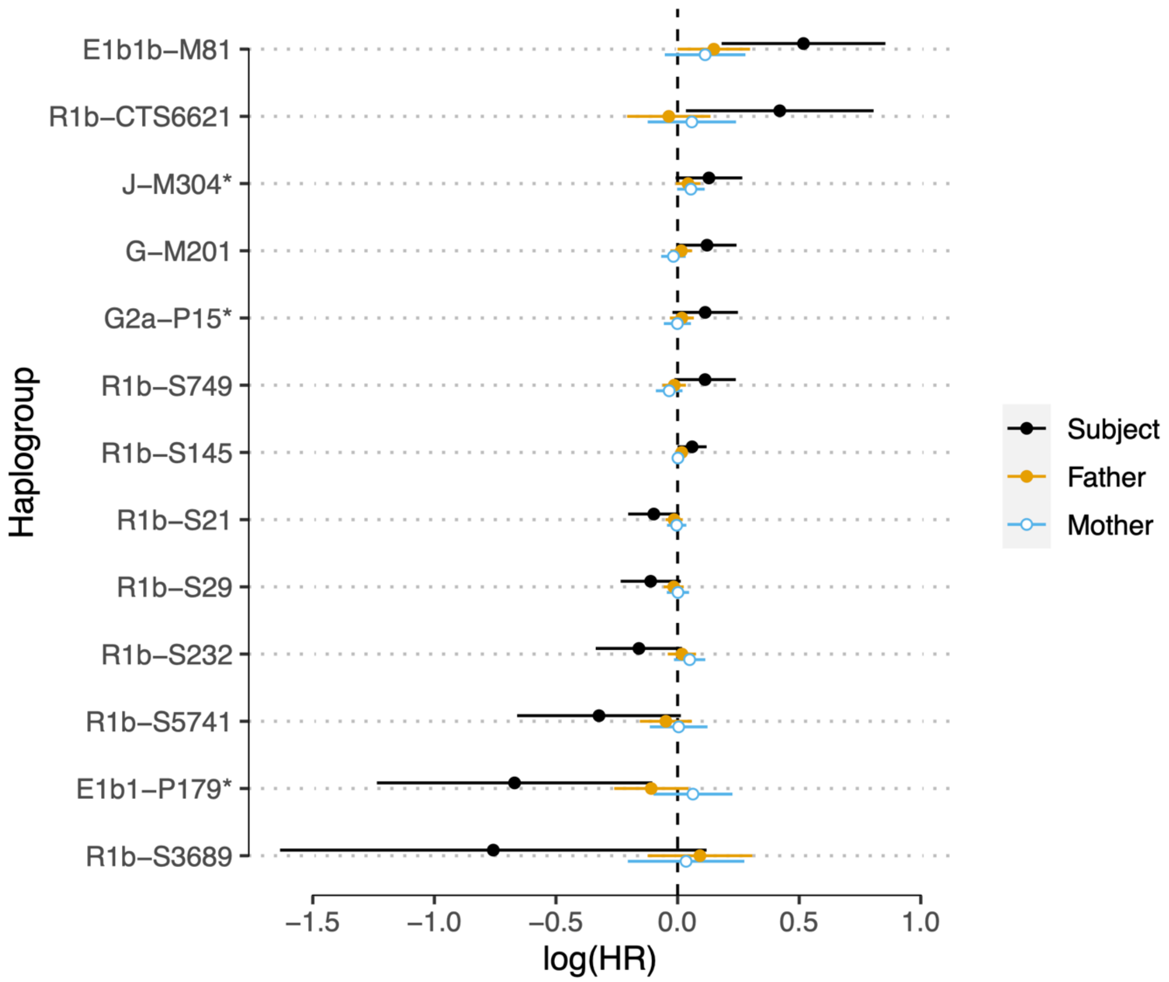

Figure S4: Parental validation of putative mortality haplogroups. Shown are MSY haplogroups with suggestive associations with all-cause mortality in White British UK Biobank subjects (P < 0.10). Units are log hazard ratios (logHR), and lines around point estimates represent 95% confidence intervals. Mother mortality is listed as a negative control. See **Table S7** for coefficients of all MSY haplogroups tested.

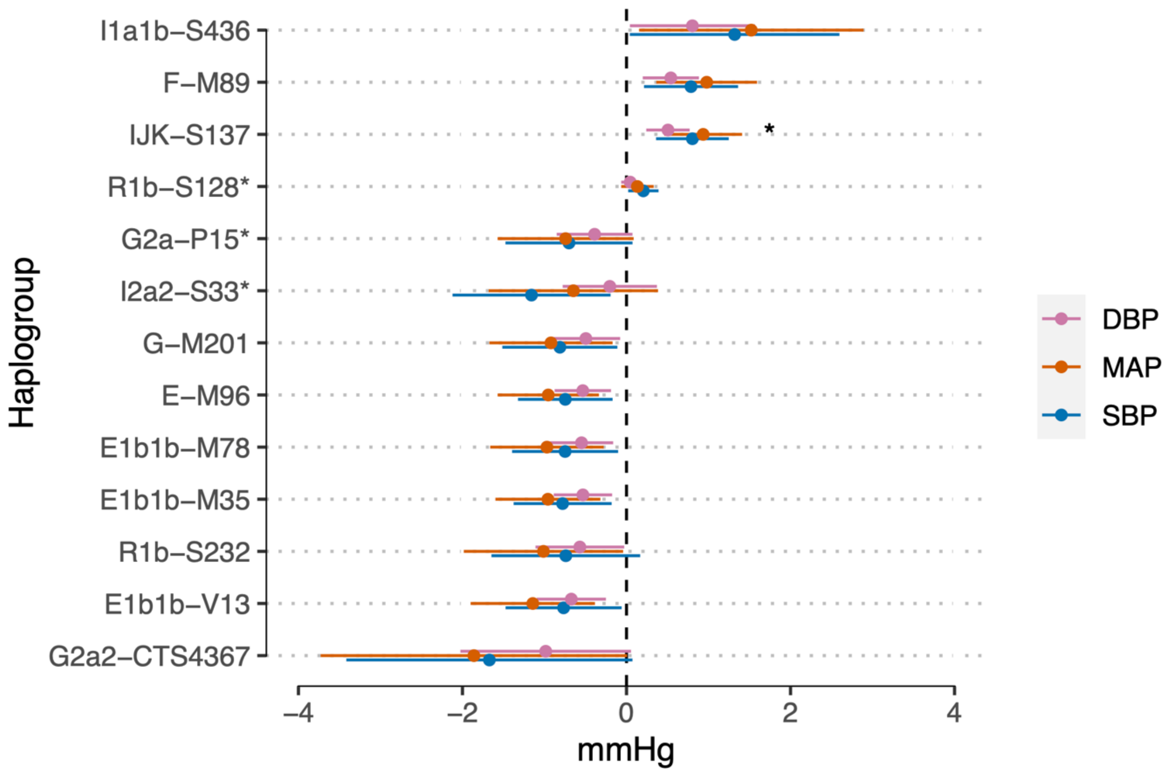

Figure S5: Effect size estimates of selected haplogroups on blood pressure traits in UK Biobank subjects. Shown are MSY haplogroups with suggestive associations ($P < 0.10$) with at least one of three blood pressure traits tested in White British UK Biobank subjects. IJK-S137 is significant after Bonferroni multiple testing corrections for 2 independent phenotypes and 38 independent haplogroups ($P < 6.58\text{x}10^{-4}$). As parental data on blood pressure traits is not available, these findings cannot be directly validated. DBP—Diastolic blood pressure. MAP—Mean arterial pressure. SBP—Systolic blood pressure. Units are mmHg, and lines around point estimates represent 95% confidence intervals. See **Table S8** for all MSY haplogroups tested.

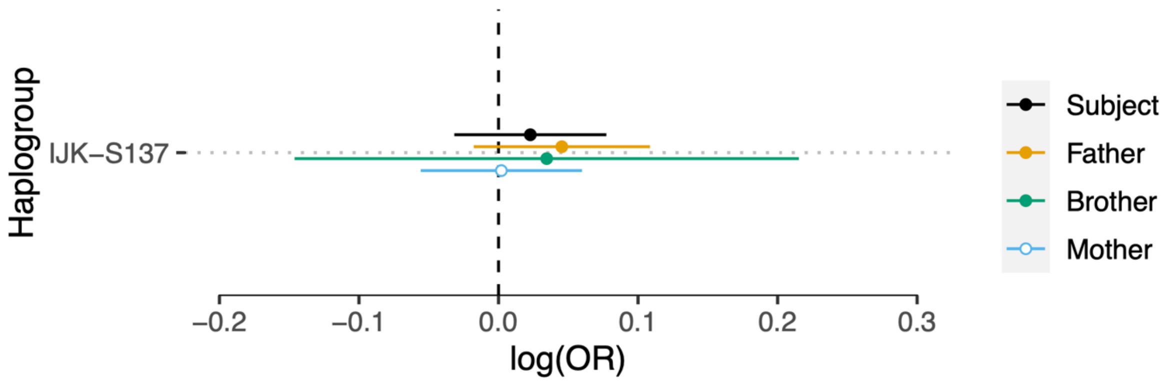

Figure S6: Effect size estimates of the IJK-S137 haplogroup on hypertension. Shown are the effect size estimates of the IJK-S137 haplogroups on self-reported hypertension in White British UK Biobank subjects and their family. Mother hypertension is listed as a negative control. Units are log odds ratios, and lines around point estimates represent 95% confidence intervals.

Major Resources Table

Animals (in vivo studies)

Species	Vendor or Source	Background Strain	Sex	Persistent ID / URL
NA				

Genetically Modified Animals

	Species	Vendor or Source	Background Strain	Other Information	Persistent ID / URL
**Parent - Male**	NA				
**Parent - Female**	NA				

Antibodies

Target antigen	Vendor or Source	Catalog #	Working concentration	Lot # (preferred but not required)	Persistent ID / URL
NA					

DNA/cDNA Clones

Clone Name	Sequence	Source / Repository	Persistent ID / URL
NA			

Cultured Cells

Name	Vendor or Source	Sex (F, M, or unknown)	Persistent ID / URL
NA			

Data & Code Availability

Description	Source / Repository	Persistent ID / URL
2011 census UK area boundaries and Townsend deprivation	UK Data Service	http://dx.doi.org/10.5257/census/aggregate-2011-2
Prevalence maps of the 90 MSY haplogroups by area of birth for UK Biobank men	Edinburgh DataShare	https://doi.org/10.7488/ds/3472
yhaplo source code used to infer MSY haplogroups	GitHub	https://github.com/23andMe/yhaplo
Statistical code used to generate the results in this study	GitHub	https://github.com/PaulTimmers/ATVB-MSY

Other

Description	Source / Repository	Persistent ID / URL
NA		
